# In vivo confocal microscopy appearance of *Fusarium* and *Aspergillus* species in fungal keratitis

**DOI:** 10.1136/bjophthalmol-2016-309656

**Published:** 2017-01-02

**Authors:** Jaya Devi Chidambaram, Namperumalsamy Venkatesh Prajna, Natasha Larke, David Macleod, Palepu Srikanthi, Shruti Lanjewar, Manisha Shah, Prajna Lalitha, Shanmugam Elakkiya, Matthew J Burton

**Affiliations:** 1 International Centre for Eye Health, London School of Hygiene and Tropical Medicine, London, UK; 2 Aravind Eye Hospital, Madurai, Tamil Nadu, India; 3 Aravind Medical Research Foundation, Madurai, Tamil Nadu, India; 4 Department of Infectious Disease Epidemiology, London School of Hygiene and Tropical Medicine, London, UK

**Keywords:** Cornea, Imaging, Infection, Microbiology, Diagnostic tests/Investigation

## Abstract

**Background:**

Clinical outcomes in fungal keratitis vary between *Fusarium* and *Aspergillus* spp, therefore distinguishing between species using morphological features such as filament branching angles, sporulation along filaments (adventitious sporulation) or dichotomous branching may be useful. In this study, we assessed these three features within Heidelberg Retina Tomograph 3 in vivo confocal microscopy (IVCM) images from culture-positive *Fusarium* and *Aspergillus* spp keratitis participants.

**Methods:**

Prospective observational cohort study in Aravind Eye Hospital (February 2011–February 2012). Eligibility criteria: age ≥18 years, stromal infiltrate ≥3 mm diameter, *Fusarium* or *Aspergillus* spp culture-positive. Exclusion criteria: previous/current herpetic keratitis, visual acuity <6/60 in fellow eye, >80% corneal thinning. IVCM was performed and images analysed for branch angle, presence/absence of adventitious sporulation or dichotomous branching by a grader masked to the microbiological diagnosis.

**Results:**

98 participants were included (106 eligible, 8 excluded as no measurable branch angles); 68 were positive for *Fusarium* spp, 30 for *Aspergillus* spp. Mean branch angle for *Fusarium* spp was 59.7° (95% CI 57.7° to 61.8°), and for *Aspergillus* spp was 63.3° (95% CI 60.8° to 65.8°), p=0.07. No adventitious sporulation was detected in *Fusarium* spp ulcers. Dichotomous branching was detected in 11 ulcers (7 *Aspergillus* spp, 4 *Fusarium* spp).

**Conclusions:**

There was very little difference in the branching angle of *Fusarium* and *Aspergillus* spp. Adventitious sporulation was not detected and dichotomous branching was infrequently seen. Although IVCM remains a valuable tool to detect fungal filaments in fungal keratitis, it cannot be used to distinguish *Fusarium* from *Aspergillus* spp and culture remains essential to determine fungal species.

## Introduction

Fungal keratitis is increasing in incidence throughout the world.^1^ In warm, humid climates such as South India, filamentous fungi are the cause of more than 60% of fungal corneal ulcers, with *Fusarium* and *Aspergillus* being the two predominant species.^2^ There is evidence that corneal healing and visual outcomes can be very different for keratitis caused by these two species; corneal infection with *Aspergillus* spp is associated with slower re-epithelialisation, increased risk of perforation and worse visual acuity at 3 months after presentation compared with *Fusarium* spp.^3^ This may in part be due to the greater susceptibility of *Fusarium* spp to natamycin, which is the main antifungal agent used to treat filamentous fungal keratitis.^4^

At present, microbiological culture of corneal scrapings remains the standard method of identifying the causative organism in fungal keratitis. Morphological features of fungal colonies and spores allow accurate identification of the fungal species, but require an experienced microbiologist and can take up to 7 days for fungi to grow and sporulate in culture.^5^ In contrast, the high-resolution imaging modality of in vivo confocal microscopy (IVCM) provides immediate visualisation of filamentous fungi within the living cornea.^6^ Previous case reports have described the IVCM appearances of filaments or hyphae of *Aspergillus* spp in patients as well as animal models of keratitis; *Aspergillus fumigatus* hyphae were reported to measure up to 3–10 microns in diameter, 200–400 microns in length and have hyphal branches emerging at 45° from the parent hyphae.^6^ ^7^ Some have postulated that the branching patterns of fungi as seen in IVCM images of keratitis could be used to differentiate fungal species. Brasnu *et al*^8^ analysed IVCM images of keratitis obtained using the Heidelberg Retina Tomograph (HRT)II confocal microscope (Heidelberg Engineering, Heidelberg, Germany) in four patients with *Fusarium* spp infection, and donor corneas infected in vitro with *A. fumigatus* and *Fusarium solani*. *Fusarium* spp filaments were reported to be 3–5 microns in diameter, 200–300 microns in length, with a hyphal branching angle of 90° in IVCM images from patients and from the infected donor cornea. For *A. fumigatus*, the branching angle in the infected donor cornea was measured as 45°.^8^ In contrast to these IVCM findings, reports of histopathological examination of fungal hyphae in tissue sections show that acute angle branching may occur in both *Fusarium* and *Aspergillus* spp.^9^ Other morphological traits have also been proposed as potential diagnostic features of certain fungi in histopathology, for example, presence of spores along hyphae (known as adventitious sporulation) in tissue samples from *Fusarium* keratitis.^10^ Also, in *Aspergillus* spp, the apical filament can directly bifurcate instead of generating side branches—this is known as dichotomous branching.^9^ To our knowledge, these features have not as yet been studied in IVCM images of fungal keratitis.

In this study, we investigated whether hyphal branching angles measured using IVCM images from patients with fungal keratitis differed between culture-positive *Fusarium* and *Aspergillus* spp corneal ulcers, and also assessed whether adventitious sporulation or dichotomous branching could be detected using IVCM.

## Materials and methods

This study was approved by the Indian Council of Medical Research, as well as the Ethics committees of Aravind Eye Care System, India and the London School of Hygiene and Tropical Medicine, UK. Tenets of the Declaration of Helsinki were adhered to in conduct of this study. Participants were enrolled after they had given their written informed consent, or a thumbprint to indicate consent in illiterate participants, witnessed by a study team member, as approved by the ethics committees.

### Study participants

Patients with clinically suspected microbial keratitis presenting to the Cornea Clinic at Aravind Eye Hospital, Madurai, Tamil Nadu (India) between February 2011 and February 2012 were assessed for eligibility to enter the study. Eligibility criteria were: age ≥18 years, corneal ulcer measuring ≥3 mm in longest diameter of stromal infiltrate and extending ≥1/3 of the corneal thickness, with an overlying epithelial defect and evidence of acute inflammation (ie, conjunctival injection and/or either anterior chamber cells, flare or hypopyon). Exclusion criteria were: previous or current herpetic keratitis, fellow eye visual acuity <6/60, descemetocele or >80% corneal thinning as assessed on slit lamp examination (due to inability to safely applanate the cornea for IVCM).

### In vivo confocal microscopy

The corneal ulcer was imaged according to a standard protocol, as previously described.^11^ The HRT3 laser scanning confocal microscope with Rostock Corneal Module (Heidelberg Engineering, Germany) was used for all IVCM imaging in this study. Briefly, the confocal microscopist applanated the IVCM onto the cornea anaesthetised with 0.5% proparacaine eye drops (Aurocaine, Aurolab, Madurai, India), and a new IVCM sterile disposable cap was used for each patient. After manually focusing, a series of volume scans was recorded at the centre and margins of the ulcer (12, 3, 6 and 9 o'clock positions), with overlapping volume scans taken from surface epithelium to the deepest region of the ulcer that could be imaged. Each volume scan had a z-stack of 40 sections, each section measuring 400 μm×400 μm (384×384 pixels) with optical thickness of 2 μm.

IVCM image sets had patient-identifying data and microbiological diagnoses removed and were allocated a random study number. All image sets were shuffled into a random order before measurements were performed.

### Branch angle measurement

Every branching hyphae present in each IVCM image within each section of each volume scan was measured by a single IVCM grader (JDC), who was masked to the microbiological diagnosis. Volume scan z-stacks were imported into ImageJ V.1 analysis software (National Institutes of Health, Bethesda, Maryland, USA) as best resolution jpeg files. For each participant, all measurable branch angles present in all section images were measured; all 40 sections in every volume scan were screened and measured for each participant. Branch angles were measured using the angle tool in ImageJ V.1. Presence or absence of dichotomous branching in all sections was also assessed. Data were recorded in Microsoft Excel for Mac 2011 (V.14.6.5).

### Microbiological culture

Immediately after IVCM imaging, corneal scrapes were taken for microbiological culture using a sterile kimura spatula after application of 0.5% proparacaine eye drops. The confocal microscopist performing the scan and the IVCM grader were masked to the microbiological culture result. Also, the microbiologists were masked to the IVCM result. Corneal scrapings obtained from the base and leading edge of the ulcer were placed directly on to blood agar and potato dextrose agar plates as well as a glass slide (for 10% potassium hydroxide staining to aid visualisation of fungal hyphae). Standard microbiological procedures were followed to identify organisms, as previously reported.^12^ Fungal culture positivity was reported if any of the following criteria were found: (a) growth of the same fungal species on ≥2 solid media or (b) semiconfluent growth at the site of inoculation in one solid medium. Fungal species were identified by morphology of the fungal colony, and lactophenol cotton blue stained hyphae and spores.^13^ Although the ocular microbiology laboratory were able to identify some but not all *Fusarium* subspecies, for this study we recorded all Fusarial growth as ‘*Fusarium* spp’, as per the European Society of Clinical Microbiology and Infectious Diseases (ESCMID) guidance, which recommends molecular typing above morphological features for accurate *Fusarium* subspecies classification.^14^

### Statistical analysis

All analyses were performed with Stata V.12.1 (StataCorp, College Station, Texas, USA). Difference in baseline demographic features between each group were compared using the Kruskal-Wallis test for non-normally distributed variables (age, symptom duration, ulcer size) and χ^2^ test for proportions (gender, prior drug use, presence of diabetes, presence of deep infiltrate). Branch angles were analysed as clustered data by patient, using a linear mixed-effects model (xtmixed) with restricted maximum likelihood estimation (reml); backwards stepwise regression was used to select predictor variables with likelihood ratio tests used for significance testing at each stage. A threshold value of p=0.10 was used for exclusion of variables, leaving fungal species, presence of a deep ulcer and prior antifungal use in the multivariate regression model, with adjustment for age and gender.

## Results

During February 2012–February 2013, 106 patients with keratitis were recruited, who were culture positive for *Fusarium* or *Aspergillus* spp*.* These patients form part of a larger cohort of 239 patients with microbial keratitis for whom we calculated the sensitivity (85.7%; 95% CI 82.2% to 88.6%) and specificity (81.4%; 95% CI 76.0% to 85.9%) of IVCM in the diagnosis of fungal keratitis compared with culture and light microscopy, full results published elsewhere.^15^ Eight patients were excluded from the IVCM analysis (5 *Fusarium* spp, 2 *Aspergillus flavus*, 1 *A. fumigatus*) due to the absence of any measurable branching hyphae in the IVCM images. In the remaining 98 participants, 68 were culture-positive for *Fusarium* spp, 24 for *A. flavus*, 4 for *A. fumigatus* and 2 for *Aspergillus terreus*. A median of 11 IVCM volume scans were obtained for each patient (range 3–28). A total of 1254 images were assessed for presence of any branching angle. Branch angles were detected and measured in 627 images in total (median 5 images measured per patient, range 1–19).

There was no significant difference between the *Fusarium* and *Aspergillus* spp groups in gender, ulcer stromal infiltrate size, presence/absence of diabetes mellitus or prior use of topical antifungals or steroid (see table 1). Participants who were culture positive for *Aspergillus* spp were older compared with those with *Fusarium* spp (median age 54 vs 45 years), and had a longer median symptom duration (7 vs 5 days, see table 1). A higher proportion of patients with *Aspergillus*-positive keratitis presented with deep ulcers involving the posterior third of the cornea compared with the *Fusarium*-positive group (80% vs 54%, see table 1). Data on topical medication used prior to presentation were available for 87 patients, of whom 45% (n=39) had used an antifungal beforehand, specifically 21% (n=18) had used a polyene antifungal (eg, natamycin), 6% (n=5) had used an azole antifungal (eg, voriconazole) and 18% (n=16) had used both drugs. Also, 10% (n=9) had used a topical steroid, 2% had used a steroid in conjunction with an antifungal. A small number of patients had a coexisting diagnosis of diabetes mellitus (n=11); evidence was very weak (p=0.068) of an association between diabetes and fungal species.

**Table 1:** Demographic data and clinical features of study participants

	*Fusarium* spp keratitis (n=68)	*Aspergillus* spp keratitis (n=30)	p Value
Median age, years (range)	45 (20–70)	54 (30–79)	0.016
Male gender, n (%)	50 (73.5)	21 (70.0)	0.719
Symptom duration, median no. of days (range)	5 (1–90)	7 (2–30)	0.003
Diabetes mellitus present, n (%)	5 (7)	6 (20)	0.068
Prior use of any antifungal, n (%)*	24 (41.4)	15 (51.7)	0.360
Prior topical steroid/ciclosporin use, n (%)*	6 (10.3)	3 (10.3)	1.000
Stromal infiltrate longest diameter, mm (range)	5.0 (3.0–10.0)	5.0 (3.0–8.3)	0.260
Presence of deep infiltrate in posterior third of cornea (assessed at slit lamp), n (%)	37 (54.4)	24 (80.0)	0.016

*For prior medication usage, n=58 for *Fusarium* group, n=29 for *Aspergillus* group (data not available for n=11).

### Branching angle of *Fusarium* spp versus *Aspergillus* spp

**Figure 1:**
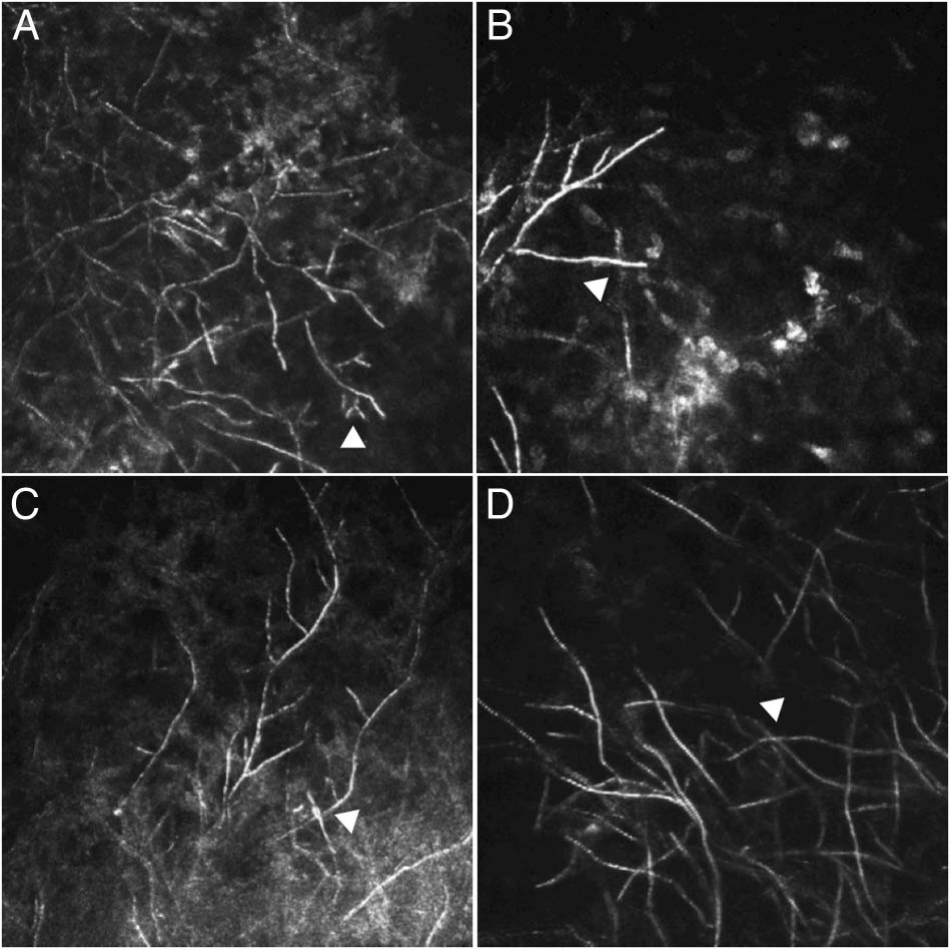
In vivo confocal microscopy (IVCM) images of keratitis caused by (A) *Aspergillus flavus* and (B), (C) and (D) *Fusarium* spp (image size 400×400 microns). All branching angles detected in all images measured <78°. Arrowhead in (A) shows dichotomous branching and in (B) shows two fungal filaments overlapping giving the false impression of a 90° branch angle. (C) and (D) show IVCM images representative of the keratitis seen in this study with arrowheads showing overlapping filaments.

The mean branching angle for filaments of *Fusarium* spp was 59.7° (95% CI 57.7° to 61.8°) and for *Aspergillus* spp was 63.3° (95% CI 60.8° to 65.8°). The small difference in branching angle between the two species did not reach significance at the 5% level in univariate analysis, but became significant when adjusting for age and gender (table 2). Figure 1 shows IVCM images from culture-positive *Fusarium* and *Aspergillus* spp ulcers, highlighting the similarity in branching structure. Branching angle from all species together were not significantly affected by symptom duration, presence of diabetes mellitus, ulcer size or prior steroid or antifungal use (table 2). Multivariate analysis including depth of ulcer, adjusting for age and gender, showed that the branching angle for *Aspergillus* spp was 4.8° greater than for *Fusarium* spp (95% CI 1.0° to 8.5°, p=0.012) and that deeper ulcers had a branching angle of 4.0° smaller than all others (95% CI −0.3° to −7.7°, p=0.034; see table 3).

**Table 2:** Effect of fungal species, sociodemographic and clinical features on fungal branching angle (analyses adjusted for age and gender)

	Change in branch angle (°)	95% CI (°)	p Value
*Aspergillus* spp (compared with *Fusarium* spp)	4.2	0.5 to 7.9	0.025
Presence of deep infiltrate in posterior third of cornea	−3.4	−7.2 to 0.3	0.075
Symptom duration (days)	−0.1	−0.3 to 0.1	0.553
Diabetes mellitus	1.2	−4.0 to 6.4	0.654
Prior steroid/ciclosporin use	1.2	−4.1 to 6.6	0.650
Stromal infiltrate diameter (mm)	−0.9	−1.9 to 0.2	0.113
Prior antifungal use	−3.1	−6.6 to 0.3	0.076

**Table 3:** Results of multivariate regression model showing effect of fungal species and ulcer depth on branch angle (adjusted for age and gender)

	Change in branch angle (°)	95% CI (°)	p Value
*Aspergillus* spp (compared with *Fusarium* spp)	4.8	1.0 to 8.5	0.012
Presence of deep infiltrate in posterior third of cornea	−4.0	−7.7 to −0.3	0.034

Among *Fusarium* spp ulcers, deep ulcers had a smaller branch angle by 4.3° (57.8° (95% CI 55.0° to 60.6°) vs 62.1° (95% CI 59.2° to 64.9°) in superficial ulcers; p=0.037). Deep *Aspergillus* spp ulcers also had smaller branch angles but the difference did not reach statistical significance (62.7° (95% CI 59.6° to 65.8°) vs 65.1° (95% CI 61.0° to 69.2°), p=0.444). Prior antifungal use also caused a significant reduction in branch angle among *Fusarium* spp, but only by 5.9° (56.6° (95% CI 52.6° to 60.6°) vs 62.5° (95% CI 60.2° to 64.9°); p=0.037). However, there was no significant effect of antifungal use on branch angle in *Aspergillus* spp (63.4° (95% CI 60.2° to 66.6°) vs no antifungal use 63.2° (95% CI 59.3° to 67.1°); p=0.99).

Only five corneal ulcers had a mean fungal branching angle ≤45°, of these four were culture positive for *Fusarium* spp and one for *A. flavus*. The remaining ulcers had mean branching angles of >45° and were positive for *Fusarium* spp (n=64) or *Aspergillus* spp (n=29). Two ulcers had at least one branch angle measured as 90° and both were caused by *Fusarium* spp.

There was a small variation in the mean branching angle within the *Aspergillus* spp, with the smallest being *A. fumigatus* at 59.1° (95% CI 53.5° to 64.6°), then *A. flavus* at 63.4° (95% CI 60.4° to 66.4°), and finally *A. terreus* at 68.9° (95% CI 58.4° to 79.5°).

### Other morphological features

Presence or absence of dichotomous branching was assessed in 92 patients (total 203 images, median 2 images per patient, range 1–10); image sets from six patients were excluded as the fungal filaments in the images were inadequate to detect this feature. Dichotomous branching was infrequently seen, detected in only seven ulcers in the *Aspergillus* spp group, compared with four in the *Fusarium* spp group (25.9% vs 6.1%).

All *Fusarium* spp ulcer images were assessed for presence of any adventitious sporulation, but this morphological feature was not detected in any of these images.

## Discussion

IVCM allows for rapid identification of fungi directly within the patient's cornea. Previous case reports have suggested that the fungal branching angle detected in IVCM imaging of keratitis can be used to differentiate between fungal species, with a 90° angle reported in *Fusarium* spp^8^ and 45° in *Aspergillus* spp.^6–8^ There is increasing evidence that keratitis caused by *Aspergillus* spp may have worse clinical outcomes, higher risk of serious complications such as corneal perforation and respond less well to natamycin therapy compared with *Fusarium* spp. Therefore, it is advantageous to be able to distinguish between the two species early on in the clinical course.^3^ ^16^

In this large prospective study, we found that *Fusarium* and *Aspergillus* spp had very similar branching angles as measured in IVCM images from patients with microbial keratitis; the difference between the two species was very small (∼4°) and became statistically significant when adjusting for age and gender. Our findings are consistent with histopathological studies, which have shown on morphological appearances alone, misdiagnosis of fungal species can occur since several fungi produce hyphae with similar morphological appearance to *Aspergillus* or *Fusarium* spp, and that in fact these two species themselves can be indistinguishable.^9^ Previous studies have compared culture results with histopathological diagnosis in tissue biopsies and have found discordant results in up to 35% of samples, particularly for *Aspergillus* and *Fusarium* spp infections. Lee *et al*^17^ found that 17% (9/53) of tissue biopsy specimens were diagnosed as *Aspergillus* spp based on histopathological appearance of acute-angle branching hyphae, but were actually culture positive for a variety of non-*Aspergillus* organisms including *Fusarium* spp, *Scedosporium* spp, *Pseudallescheria* spp, *Phialophora verrucosa* and *Trhichophyton* spp. Schofield *et al*^18^ found that in 35% (8/23) tissue biopsies from infected burn wounds that were classified by histopathology as having hyphae consistent with *Aspergillus* spp, the causative organism isolated in culture was actually *Fusarium* spp, *Trichosporon* spp, *Curvularia* spp or *Candida* spp. A further study reported misclassification of biopsy specimens in 21% of cases (n=10/47), including three cases reported as *Aspergillus* spp on histopathology but culture positive for *Fusarium* spp, *Scedosporium* spp or *Rhizopus* spp.^19^ Since such misclassifications may have an impact on starting correct antimicrobial therapy, recent guidelines recommend using both histopathology and culture results together to make the diagnosis of invasive aspergillosis and fusariosis.^14^ ^20^

Fungal filament appearance, branching angle and growth can be affected by several factors, including topography of the host tissue,^21^ local availability of oxygen or nutrients (eg, glucose), tendency to grow away from neighbouring hyphae (known as negative autotropism) as well as health of the fungal cell wall and cell membrane.^22^ Both polyene and the azole antifungals act on ergosterol, a major component of the fungal cell membrane, to disrupt hyphal growth.^23^ ^24^ Both *Fusarium* and *Aspergillus* spp had smaller branch angles when treated with antifungal prior to presentation or in deep ulcers but only reaching statistical significance for *Fusarium* spp. However, the magnitude of this effect (ie, <6° at most) was very small and therefore may not have masked any true difference in branch angle between the two species. Further studies are therefore required to more fully explore the impact of different antifungals on fungal morphology.

In addition to branch angle, other characteristic morphological features have been noted in culture and/or histopathology for some keratitis-causing fungi. *Fusarium*, *Acremonium* and *Paecilomyces* spp can sometimes develop spores within the infected tissue, known as adventitious sporulation, and this has also been detected in keratitis.^10^ In our series of *Fusarium*-positive ulcers, we were unable to detect adventitial sporulation. This may be due to the size of the fungal spores reaching the limits of resolution of the laser scanning HRT confocal microscope (ie, 2 microns laterally).^13^ ^25^ *Aspergillus* spp have been described to have dichotomous branching, where the tip of the distal fungal hyphae splits into two branches.^9^ Although we did detect this phenomenon in a higher proportion of ulcers positive for *Aspergillus* spp than in the *Fusarium* spp, further studies with a larger sample of *Aspergillus* keratitis are required to confirm this finding.

This study had several limitations. The IVCM images obtained in this study were two-dimensional and therefore the three-dimensional (3D) structure of the fungal mycelium growing within the entire cornea could not fully be appreciated. Advances in IVCM imaging may allow 3D reconstruction of HRT3/Rostock Corneal Module images of the entire cornea in the future,^25^ thus enabling research into the relationship between hyphal growth, branching and tissue topography. We found that the majority of ulcers in the study were culture positive for *Fusarium* spp. This pattern of increased incidence of keratitis due to *Fusarium* spp in this geographical region has been previously reported.^2^ Participants with culture-positive *Aspergillus* spp ulcers more frequently had a longer symptom duration and deeper corneal involvement at presentation, but all of these parameters still only impacted on branch angle by a few degrees.

In summary, we have found very little difference between the hyphal branching angle in IVCM images taken from culture-positive *Fusarium* and *Aspergillus* spp ulcers. Although IVCM remains a valuable tool to detect fungal filaments in corneal ulcers, it cannot be used to distinguish *Fusarium* spp from *Aspergillus* spp and culture remains essential to determine fungal species.
